# The Role of +4U as an Extended Translation Termination Signal in Bacteria

**DOI:** 10.1534/genetics.116.193961

**Published:** 2016-11-29

**Authors:** Yulong Wei, Xuhua Xia

**Affiliations:** * Department of Biology, University of Ottawa, Ontario K1N 6N5, Canada; † Ottawa Institute of Systems Biology, Ontario K1H 8M5, Canada

**Keywords:** translation termination, termination read-through, gene expression, release factors

## Abstract

Termination efficiency of stop codons depends on the first 3′ flanking (+4) base in bacteria and eukaryotes. In both *Escherichia coli* and *Saccharomyces cerevisiae*, termination read-through is reduced in the presence of +4U; however, the molecular mechanism underlying +4U function is poorly understood. Here, we perform comparative genomics analysis on 25 bacterial species (covering Actinobacteria, Bacteriodetes, Cyanobacteria, Deinococcus-Thermus, Firmicutes, Proteobacteria, and Spirochaetae) with bioinformatics approaches to examine the influence of +4U in bacterial translation termination by contrasting highly- and lowly-expressed genes (HEGs and LEGs, respectively). We estimated gene expression using the recently formulated Index of Translation Elongation, I_TE_, and identified stop codon near-cognate transfer RNAs (tRNAs) from well-annotated genomes. We show that +4U was consistently overrepresented in UAA-ending HEGs relative to LEGs. The result is consistent with the interpretation that +4U enhances termination mainly for UAA. Usage of +4U decreases in GC-rich species where most stop codons are UGA and UAG, with few UAA-ending genes, which is expected if UAA usage in HEGs drives up +4U usage. In HEGs, +4U usage increases significantly with abundance of UAA nc_tRNAs (near-cognate tRNAs that decode codons differing from UAA by a single nucleotide), particularly those with a mismatch at the first stop codon site. UAA is always the preferred stop codon in HEGs, and our results suggest that UAAU is the most efficient translation termination signal in bacteria.

DIFFERENT stop codons have different termination efficiency, and replacing UGA with UAA reduces termination read-through of human genes expressed in *Escherichia coli* (Meng *et al.* 1995; Cesar Sanchez *et al.* 1998). The discrepancies in termination efficiency among stop codons in bacteria are largely attributed to: (1) the competition between near-cognate transfer RNAs (tRNAs) (nc_tRNAs) and class I release factors (RF1 and RF2) in decoding stop codons (Nakamura *et al.* 1996; Tate *et al.* 1999; Blanchet *et al.* 2014), mediated by the relative abundance of RF1 and RF2 (Korkmaz *et al.* 2014; Wei *et al.* 2016), and (2) nucleotide sites downstream of stop codons interacting with 18S ribosomal RNA (rRNA) and modulating the structural stability of binding sites for RF1 and RF2 (Namy *et al.* 2001) or interacting directly with release factors based on inferences from cross-linking experiments in both bacterial (Poole *et al.* 1998) and eukaryotic species (Bulygin *et al.* 2002).

Termination efficiency of stop codons depends on the first 3′-flanking (+4) base in bacterial species such as *E. coli* and *Salmonella typhimurium* (Bossi and Ruth 1980; Miller and Albertini 1983; Tate *et al.* 1995; Poole *et al.* 1998) and in eukaryotes (Manuvakhova *et al.* 2000; Jungreis *et al.* 2011). The inefficiency of translation termination associated with +4C, especially in UGA-ending genes, is well-documented in both bacteria (Brown and Tate 1994; Poole *et al.* 1995; Tate *et al.* 1999) and eukaryotes (Manuvakhova *et al.* 2000; Namy *et al.* 2001; Jungreis *et al.* 2011; Dabrowski *et al.* 2015; Beznoskova *et al.* 2016). UGA-C contributes to the autoregulation of *prfB* (coding RF2) translation (Craigen *et al.* 1985; Craigen and Caskey 1986), with a truncated RF2 produced when functional RF2 is abundant and a full-length functional RF2 produced when functional RF2 is rare. Baranov *et al.* (2002) identified the *prfB* gene in 87 bacterial species using BLAST (Altschul *et al.* 1990), and revealed programmed frameshift in 70% of these bacteria. The segment involved in the frameshift (CUU UGA CNN) and the translated segment (CUU GAC NNN) are always conserved, showing ribosome slippage at UGA-C.

UGA is particularly prone to be misread by tRNA^Trp^ when followed by +4A in *E. coli* (Engelberg-Kulka 1981) and yeast (Geller and Rich 1980). A recent study in yeast by Beznoskova *et al.* (2016) measured the read-through of termination tetranucleotides (*e.g.*, UGA-C) in dual luciferase constructs. Indeed, +4C increases read-through in all three stop codons, but particularly so in UGA in yeast. Furthermore, UGA-A and UGA-G enhance misreading by tRNA^Trp^ and tRNA^Cys^, respectively (Beznoskova *et al.* 2016). In contrast, UGA-U, UAA-U, and UAG-U are all associated with low read-through (Beznoskova *et al.* 2016). The finding that +4U reduces termination read-through is consistent with the observation that this base is overrepresented in *E. coli*, especially in UAA-ending genes (Brown *et al.* 1990; Poole *et al.* 1995; Tate *et al.* 1996).

Early studies in *E. coli* suggest that the decoding efficiency of RF2 depends on the +4 base (Brown and Tate 1994; Tate *et al.* 1996; Poole *et al.* 1997, 1998). In particular, Brown and Tate (1994) and Poole *et al.* (1998) revealed that RF2 cross-links with UAA, and with UGA at the first (+ 1) base and the downstream +4 base; and the cross-link efficiency between RF2 and stop codons is promoted in the presence of +4U. Thus, +4U may participate in recruiting RF2 to the stop codon. Similarly, studies in eukaryotes found cross-linking between the +4 base and eRF1 in human UAA-ending genes (Bulygin *et al.* 2002).

If the +4 site really serves as part of an extended stop signal, and if +4U enhances the stop signal relative to other nucleotides, then one can immediately predict that highly-expressed genes (HEGs), which are under selection to evolve toward high translation initiation, elongation, and termination efficiency, should prefer +4U more strongly than lowly-expressed genes (LEGs). Furthermore, it is possible that different stop codons may require different +4 nucleotides to enhance the stop signal. In particular, GC-rich species may have difficulty maintaining a +4U site and may have different combinations of stop codons and +4 nucleotides from those AT-rich species. Testing these predictions constitutes the first part of this paper.

Stop codons can be misread by nc_tRNAs in *E. coli* (Sambrook *et al.* 1967; Strigini and Brickman 1973), coliphage (Weiner and Weber 1973), eukaryotic viruses (Beier and Grimm 2001), the yeast *Saccharomyces cerevisiae* (Blanchet *et al.* 2014), and mammals (Geller and Rich 1980). Available data suggest termination read-through is most frequent at UGA, less at UAG, and least at UAA, in both bacteria and eukaryotes (Parker 1989; Jorgensen *et al.* 1993; Tate *et al.* 1999; Dabrowski *et al.* 2015).

Stop codon read-through can occur in yeast by the incorporation of nc_tRNAs with wobble-pairing at the third stop codon site (Beznoskova *et al.* 2015, 2016), or at the first stop codon site involving tRNA^UUG/Gln^ and tRNA^CUG/Gln^ misreading UAA and UAG, respectively (Blanchet *et al.* 2014; Roy *et al.* 2015, 2016). In the yeast, tRNA^Gln^, tRNA^Tyr^, and tRNA^Lys^ can misread stop codons UAA and UAG, whereas tRNA^Trp^, tRNA^Cys^, and tRNA^Arg^ can misread stop codon UGA (Blanchet *et al.* 2014). Misreading of UAA and UAG by tRNA^Gln^ also occurs in *E. coli* (Nilsson and Ryden-Aulin 2003). UGA can be misread by tRNA^Trp^ decoding UGG in both *E. coli* and *Bacillus subtilis* (Engelberg-Kulka 1981; Matsugi and Murao 1999, 2000; Nilsson and Ryden-Aulin 2003).

How +4U may enhance the stop codon signal remains unknown. Namy *et al.* (2001) speculated that, in yeast UAG-ending genes, several bases at the 3′-UTR leading with +4C may pair with yeast 18S rRNA and destabilize secondary structures in the ribosome, preventing release factors from binding to stop codons. However, it is possible that +4U may serve to prevent misreading of stop codons by nc_tRNA. If this is the case, then + U usage should increase with the frequency of nc_tRNA, which is an easily testable prediction. Testing this prediction constitutes the second part of this study.

We analyzed the genomic and proteomic data in 25 bacterial species (Supplemental Material, Table S1 in File S2), whose protein abundance data are present in PaxDB 4.0 (Wang *et al.* 2015), to examine the effect of the +4 site and nc_tRNA on termination efficiency of the three stop codons. We found that +4U was consistently overrepresented in HEGs in contrast to LEGs in bacteria. However, +4U usage in HEGs decreased in GC-rich bacterial species where most stop codons are UGA and UAG, suggesting that UGA and UAG do not need +4U as a stop signal enhancer as much as UAA. In HEGs, +4U usage also increases significantly with the abundance of UAA nc_tRNAs, suggesting that +4U increases UAA termination efficiency, presumably by reducing the misreading of UAA by nc_tRNAs.

## Materials and Methods

### Protein expression data

Proteomic data are available in PaxDB 4.0 (Wang *et al.* 2015) for 26 bacterial species of which one (*Mycoplasma pneumoniae*) is excluded from this study. The reason for the exclusion is that *M. pneumoniae* uses genetic code 4, thus is different from the other species which use genetic code 11. *M. pneumoniae* uses only two stop codons (UAA and UAG, decoded by RF1) and does not have *prfB* genes coding for RF2 (which would decode UAA and UGA). The integrated data set was chosen when there were multiple data sets for a single species. *B. subtilis* protein IDs in PaxDB are UniProt IDs; the “Retrieve/ID mapping” function in UniProt (Pundir *et al.* 2016) was used to map UniProt IDs to Gene IDs.

Proteomic data are used to classify genes into HEGs and LEGs for compiling codon usage tables of HEGs and LEGs that are needed for computing the index of translation elongation or I_TE_ (Xia 2015). I_TE_ incorporates the tRNA-mediated selection and the effect of background mutation, and is therefore advantageous over codon adaptation index (Sharp and Li 1987; Xia 2007) or tRNA adaptation index (dos Reis *et al.* 2004) when genomes of diverse GC% are used in analysis. We used I_TE_ as a proxy of translation efficiency. That is, genes with a high I_TE_ are expected to be under stronger selection for translation efficiency than genes with a low I_TE_.

For each of the 25 species, 40 ribosomal protein genes with the highest protein abundances (parts per million) and 40 genes with the lowest nonzero protein abundances were taken from each species to compile codon usage for HEGs and LEGs, respectively. I_TE_ was computed with the option of “Break 8-fold and 6-fold families into 2.” Only nonpseudo and nonhypothetical genes were selected in this study.

Among the 25 species, five species (*Bartonella henselae*, *Helicobacter pylori*, *Leptospira interrogans*, *Pseudomonas aeruginosa*, and *Synechocystis sp*.) do not exhibit clear differences in codon usage between HEGs and LEGs. This means that I_TE_ will not be a good proxy for translation efficiency in these five species. *Shigella flexneri* is phylogenetically nested within *E. coli* strains and therefore does not supply an independent data point. For this reason, only those 19 remaining species were used for I_TE_-related analysis.

### Processing bacterial genomes

The bacterial genomes were retrieved from GenBank, and coding sequences (CDSs) were extracted by using DAMBE (Xia 2013b) for computing I_TE_. An alternative set of HEGs consists of all ribosomal protein genes extracted from DAMBE (Xia 2013b) based on genomic annotation. We also extracted small subunit rRNA (ssu rRNA) genes from each species for building a phylogenetic tree for computing independent contrasts. For each stop codon, their nc_tRNAs (Table 1) were compiled. No tRNA had anticodons AUA or ACA in the species studied and, thus, they are not included in Table 1.

**Table 1:** Anticodons of nc_tRNAs for each of the three stop codons

UAA	UAG	UGA
Glu-TTC	Glu-CTC	Gly-TCC
Gln-TTG	Gln-CTG	Arg-TCG
Lys-TTT	Lys-CTT	Arg-TCT
Leu-TAA	Leu-CAA	Leu-TAA
Ser-TGA	Trp-CCA	Ser-TGA
Tyr-GTA	Ser-CGA	Trp-CCA
	Tyr-GTA	Cys-GCA

No tRNA (transfer RNA) has AUA or ACA anticodons in all of the bacterial species we studied.

### Phylogenetic reconstruction and independent contrasts

**Figure 1:**
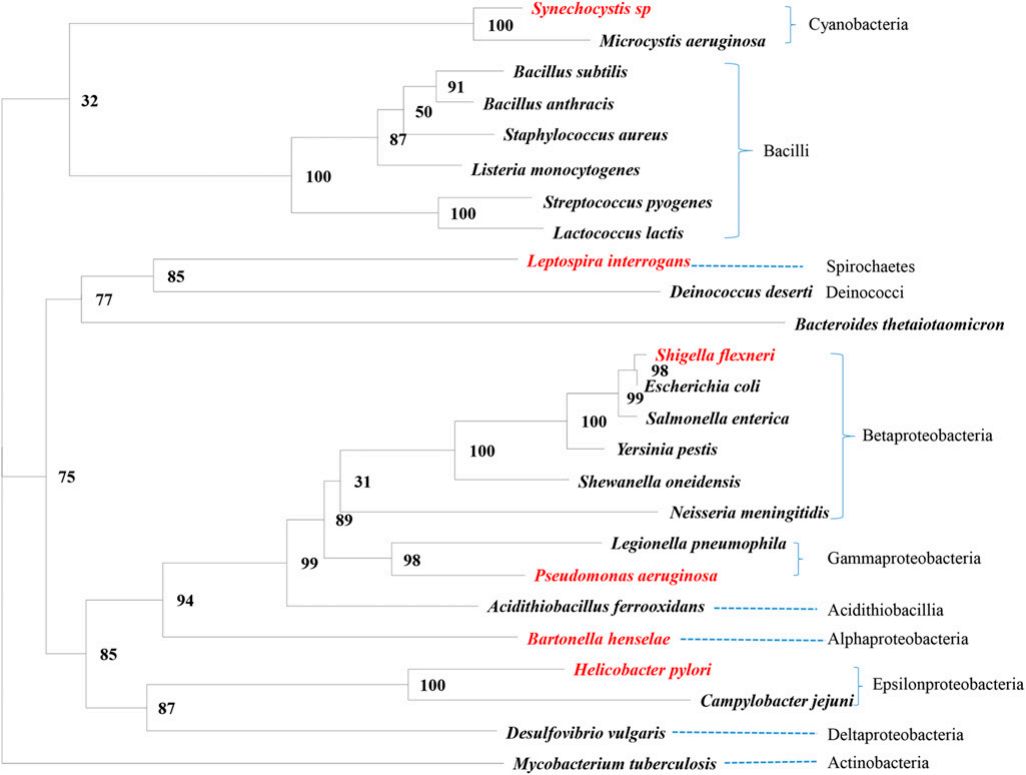
Phylogenetic relationship among the 25 bacterial species. The six species in red were not used in I_TE_-related analysis (see *Materials and Methods* for reason of exclusion). The branch length for *Bacteroides thetaiotaomicron* was shortened by nearly one-third for a more compact display. I_TE_, Index of Translation Elongation.

Variables measured from a set of species are typically not independent because of shared ancestry. Phylogeny-based independent contrasts (Felsenstein 1985) were computed to alleviate this problem. We aligned ssu rRNA sequences aligned by MAFFT (Katoh *et al.* 2009) with the LINSI option, which generates the most accurate alignment (“–localpair” and “–maxiterate = 1000”). PhyML (Guindon and Gascuel 2003) was used for phylogenetic reconstruction, with general time reversible (GTR) substitution model and six categories of gamma-distributed rates. The resulting tree (Figure 1) was used for computing independent contrasts (Felsenstein 1985), as numerically illustrated in Xia (2013a). The same approach was used to reconstruct the tree for the 19 species (indicated in Figure 1) in I_TE_-related analysis.

Because the bacterial species involve deep phylogeny with limited resolution close to the root node, we assessed the effect of different trees on the results of independent contrasts by using 100 bootstrapped trees. DAMBE takes a file with the 100 trees and automatically performs independent contrasts for each tree. We have also used a tree built with PhyPA, suitable for deep phylogenetic relationships (Xia 2016). The PhyPA is based on pairwise sequence alignment using default option simultaneously estimated distances based on a TN93 model (Tamura and Nei 1993).

### Data availability

**Figure 2:**
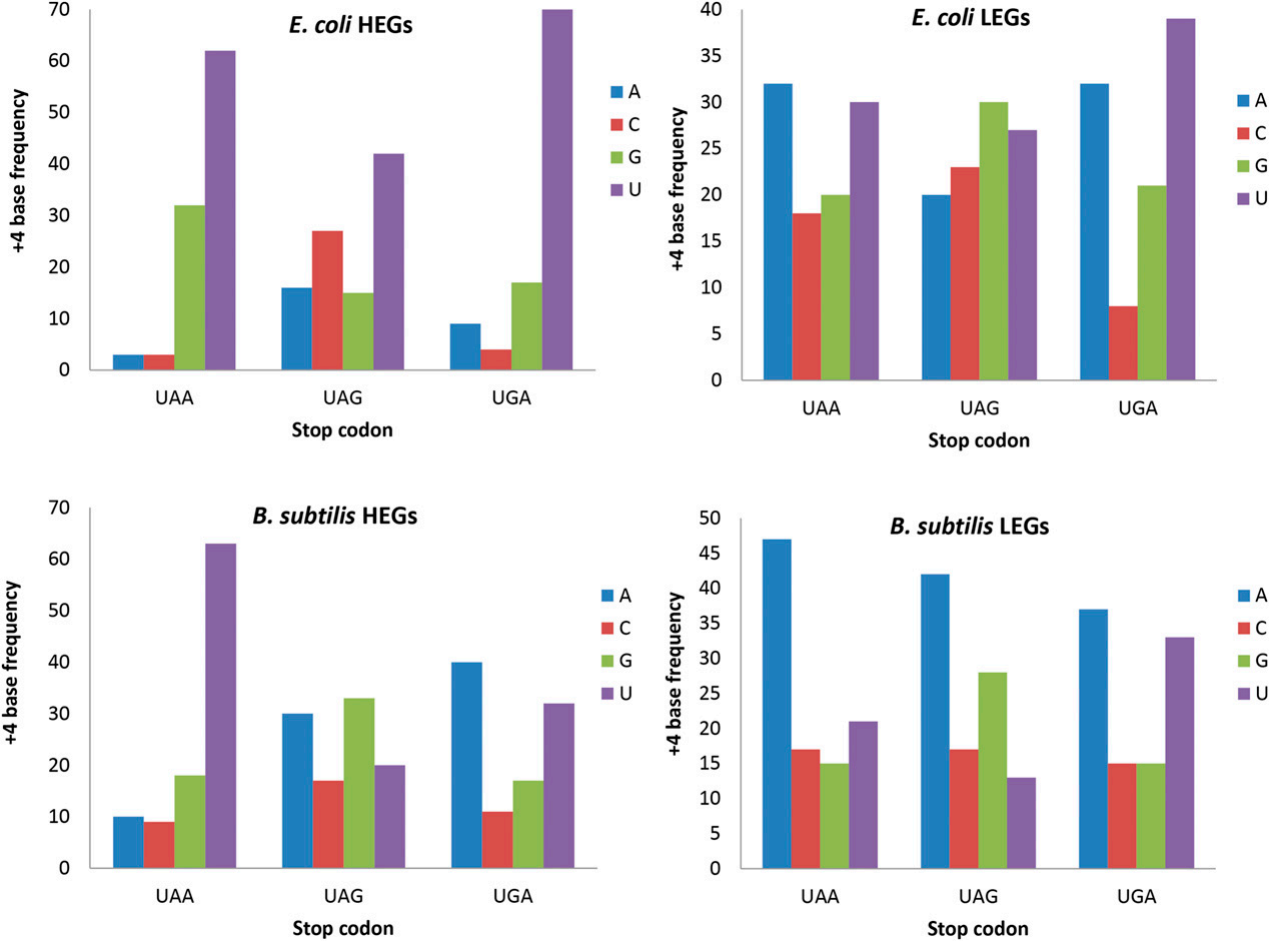
Relationship between +4 nucleotide usage and stop codons in *E. coli* and *B. subtilis*, contrasting between 100 highly- and 100 lowly-expressed genes (HEGs and LEGs, respectively) for each stop codon, respectively. Only nonpseudo and nonhypothetical genes are used.

**Figure 3:**
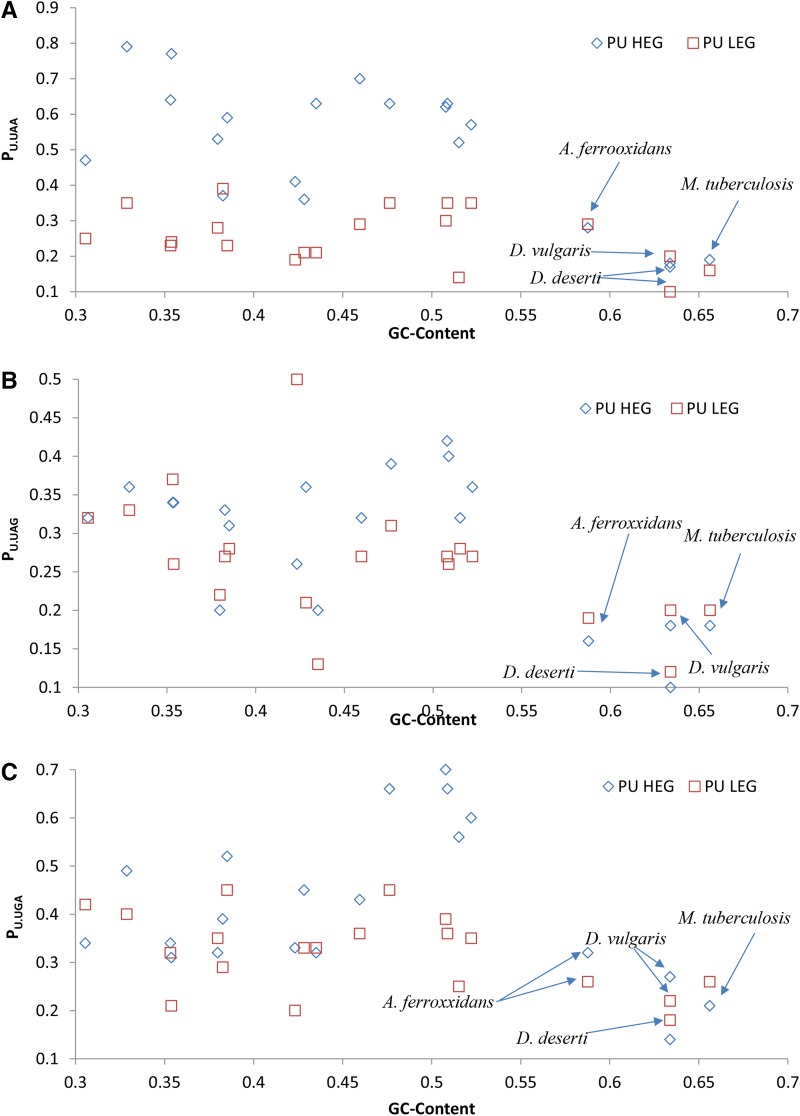
Relationship between genomic GC-content (proportion of G and C in the genome) and +4U usage measured as the proportion of +4U at the +4 site and designated by P_U.UAA_ (A), P_U.UAG_ (B), and P_U.UGA_ (C), respectively, for the three stop codons in 19 bacterial species. 100 HEGs and 100 LEGs are used for each stop codon. Only nonpseudo, nonhypothetical genes are used. The four species with high GC-contents (> 58%) are indicated. HEGs, highly-expressed genes; LEGs, lowly-expressed genes.

**Figure 4:**
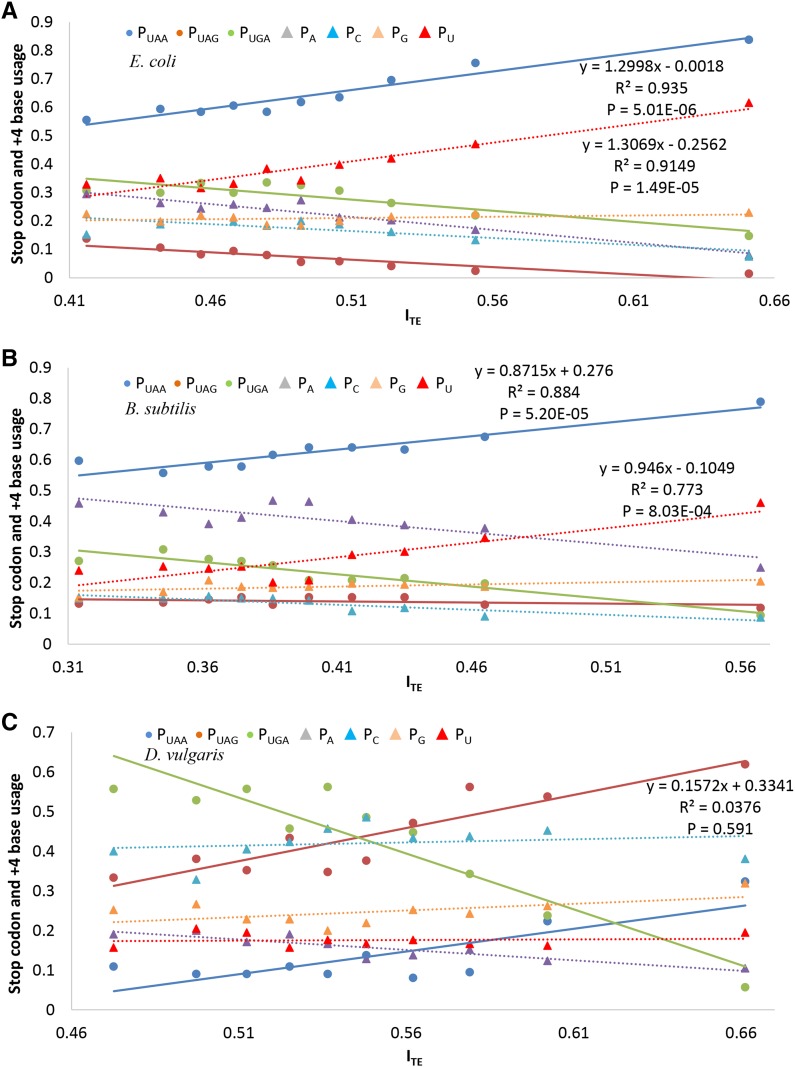
Relationship between I_TE_ and usage of termination signals (stop codons and +4 bases), in *E. coli* (A), *B. subtilis* (B), and *D. vulgaris* (C). All nonpseudo, nonhypothetical CDSs were ranked by I_TE_ and binned into 10 sets; the stop codon usage and +4 base usage was obtained in each set. Stop codon usage (P_UAA_, P_UAG_, and P_UGA_) is represented by solid lines; +4 base usage (P_A_, P_C,_ P_G_, and P_U_) is represented by dotted lines. CDSs, coding sequences; I_TE_, Index of Translation Elongation.

**Figure 5:**
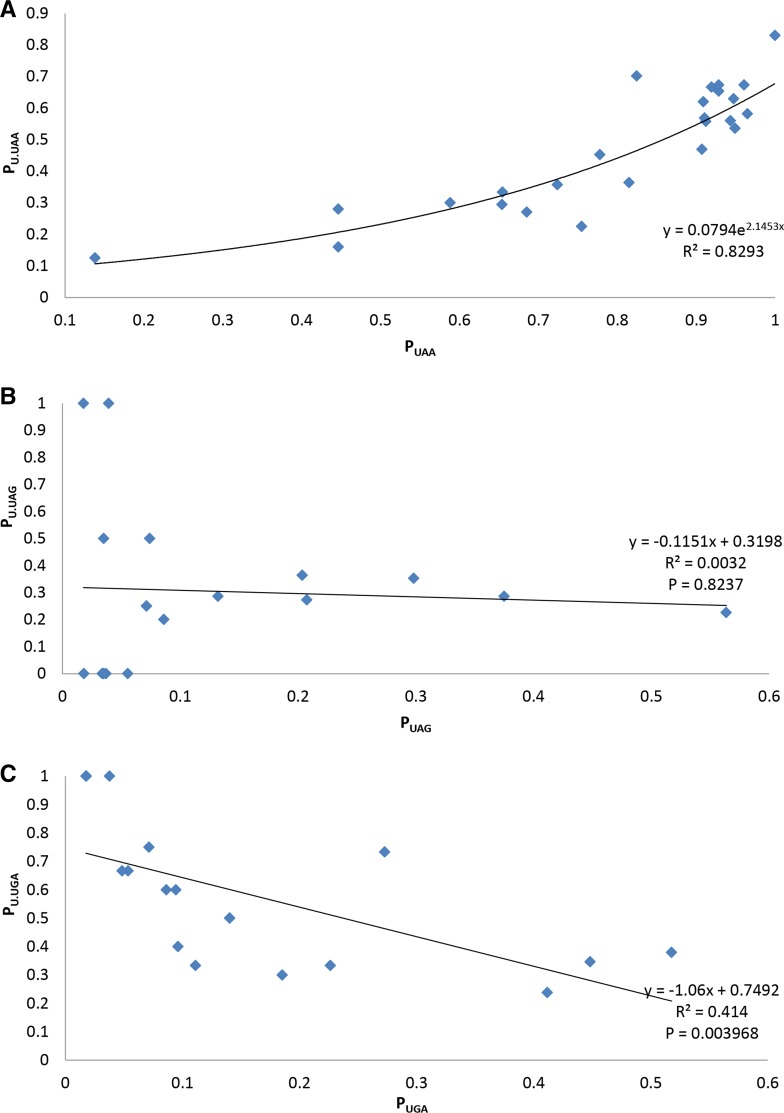
Relationship between stop codon and +4 base usage, represented with regression between the proportions of stop codons (P_UAA_, P_UAG_, and P_UGA_) and proportion of their +4U (P_U.UAA_, P_U.UAG_, and P_U.UGA_), and shown in (A), (B), and (C), respectively. Data from all 30S and 50S ribosomal protein genes in 25 bacterial species, excluding the data point if the stop codon usage is zero.

**Figure 6:**
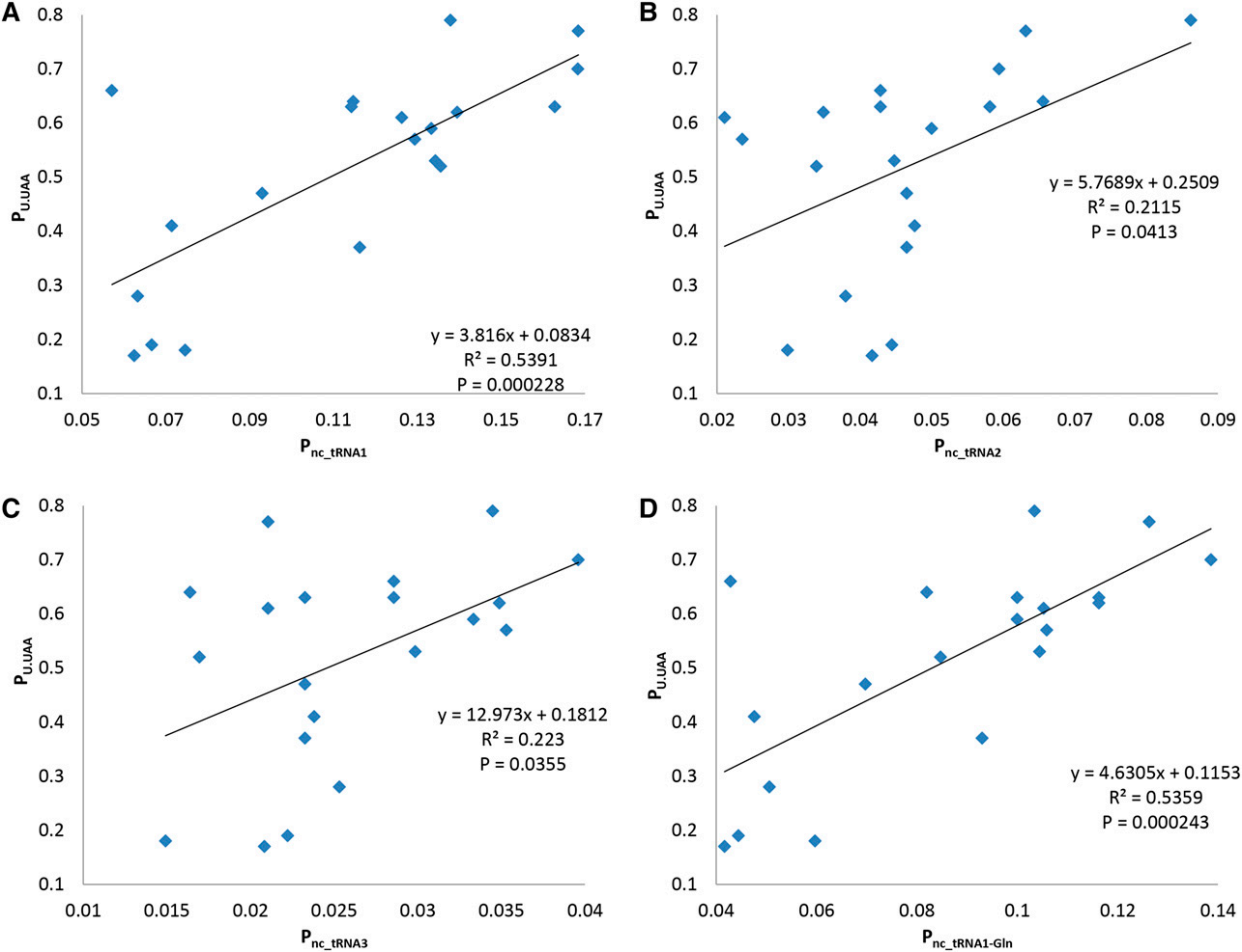
Relationship between nc_tRNA abundance and +4U usage, represented by linear regression between 100 UAA-ending HEGs (highest I_TE_ scores) and abundance of UAA nc_tRNAs with a single mismatch at (A) the first stop codon site, (B) the second stop codon site, (C) the third stop codon site, and (D) the first stop codon site, omitting tRNA^Gln^, 5′-TTG-3′, in 19 bacterial species. CDSs, coding sequences; HEGs, highly-expressed genes; I_TE_, Index of Translation Elongation; nc_tRNA, near-cognate tRNA; tRNA, transfer RNA.

File S1 contains a detailed description of File S2, File S3, File S4, and File S5, and provides additional details for the *Materials and Methods*. File S2 contains supplementary tables and figures discussed in this manuscript. HEG_RSCU and LEG_RSCU data are provided in File S3. File S4 contains I_TE_ scores for all CDSs in 19 bacterial species. File S5 contains the associated data for Figure 2, Figure 3, Figure 4, Figure 5, and Figure 6.

## Results

### HEGs and LEGS differ in the relationship between +4U and stop codons

+4U is strongly overrepresented in all stop codons in *E. coli*, especially for UAA-ending and UGA-ending HEGs (Figure 2). In contrast, +4U is overrepresented in UAA-ending HEGs relative to UAA-ending LEGs in *B. subtilis* (Figure 2). In each species, the nucleotide distribution at the +4 site depends significantly on stop codons (*P* < 0.0001) when tested by log-linear models. The difference between the two species is also highly significant (*P* < 0.0001), with the main contribution to the difference from +4 sites following UAG and UGA. While both species exhibit overuse of +4U in UAA-ending HEGs, only *E. coli* overused +4U in UGA-ending HEGs. A previous experimental study demonstrated a strong effect of +4U in increasing the termination efficiency of UGA (Kopelowitz *et al.* 1992).

All five of the species belonging to Betaproteobacteria (Figure 1) share the *E. coli* pattern, *i.e.*, +4U overrepresented in all stop codons in HEGs relative to LEGs (Table 2), and all seven species belonging to Cyanobacteria and Bacilli share the *B. subtilis* pattern, with strong overrepresentation of +4U in UAA-ending HEGs relative UAA-ending LEGs, but no clear pattern involving UAG and UGA codons (Table 2). Species with the *E. coli* pattern generally have far more RF2 than RF1, whereas those with the *B. subtilis* pattern have more RF1 than RF2 (Wei *et al.* 2016). It is likely that +4U increases termination efficiency for RF2 decoding UAA and UGA, whereas RF1 may benefit from +4U only in decoding UAA. This would suggest that overuse of UAA by HEGs would result in overuse of +4U. This is indeed the case. The species with overrepresented +4U in HEGs, *i.e.*, the seven species belonging Cyanobacteria and Bacilli and the five species belonging to Betaproteobacteria, indeed all have more UAA overrepresented in HEGs than LEGs.

**Table 2:** The usage of +4U (P_U_) in 100 nonpseudo and nonhypothetical UAA, UAG, and UGA-ending HEGs and LEGs, ranked by I_TE_, in 19 bacterial species, together with the species’ accession number and genomic GC content

Species Name	Accession	GC%	UAA		UAG		UGA	
			P_U.HEG_	P_U.LEG_	P_U.HEG_	P_U.LEG_	P_U.HEG_	P_U.LEG_
*Microcystis aeruginosa*	NC_010296	42.331	0.41	0.19	0.26	0.5	0.33	0.2
*Bacillus anthracis*	NC_005945	35.379	0.77	0.24	0.34	0.26	0.31	0.21
*Bacillus subtilis*	NC_000964	43.514	0.63	0.21	0.2	0.13	0.32	0.33
*Staphylococcus aureus*	NC_002758	32.878	0.79	0.35	0.36	0.33	0.49	0.40
*Listeria monocytogenes*	NC_003210	37.981	0.53	0.28	0.2	0.22	0.32	0.35
*Streptococcus pyogenes*	NC_002737	38.512	0.59	0.23	0.31	0.28	0.52	0.45
*Lactococcus lactis*	NC_002662	35.329	0.64	0.23	0.34	0.37	0.34	0.32
*Deinococcus deserti*	NC_002937	63.388	0.17	0.10	0.10	0.12	0.14	0.18
*Bacteroides thetaiotaomicron*	NC_004663	42.837	0.66	0.33	0.36	0.21	0.45	0.33
*Escherichia coli*	NC_000913	50.791	0.62	0.3	0.42	0.27	0.7	0.39
*Salmonella enterica*	NC_003197	52.222	0.57	0.35	0.36	0.27	0.6	0.35
*Yersinia pestis*	NC_003143	47.636	0.63	0.35	0.39	0.31	0.66	0.45
*Shewanella oneidensis*	NC_004347	45.961	0.7	0.29	0.32	0.27	0.43	0.36
*Neisseria meningitidis*	NC_003112	51.528	0.52	0.14	0.32	0.28	0.56	0.25
*Legionella pneumophila*	NC_002942	38.27	0.37	0.39	0.33	0.27	0.39	0.29
*Acidithiobacillus ferrooxidans*	NC_011761	58.773	0.28	0.29	0.16	0.19	0.32	0.26
*Campylobacter jejuni*	NC_002163	30.549	0.47	0.25	0.32	0.32	0.34	0.42
*Desulfovibrio vulgaris*	NC_002937	63.388	0.18	0.2	0.18	0.2	0.27	0.22
*Mycobacterium tuberculosis*	NC_000962	65.615	0.19	0.16	0.18	0.2	0.21	0.26

A value of 0.26 under UAA/P_U.HEG_ means 26 genes out of 100 UAA-ending HEGs have +4U. Horizontal lines delineate major taxonomic groups corresponding to Figure 1. HEG, highly-expressed gene; LEG, lowly-expressed gene.

The usage of +4U changes with genomic GC% (Figure 3), with the overuse of +4U most pronounced in UAA-ending genes with the proportion of genomic GC from low to slightly higher than 50% (Figure 3). Based on the Wilcoxon rank sum test with continuity correction, the difference in +4U usage between HEGs and LEGs is significant in UAA-ending genes (*P* = 0.000327, two-tailed test), but not significant in UAG-ending genes (*P* = 0.2538, two-tailed test) and UGA-ending genes (*P* = 0.0795, two-tailed test). However, four species with high genomic GC contents (> 58.7%) (*M. tuberculosis*, *Deinococcus deserti*, *Desulfovibrio vulgaris*, and *Acidithiobacillus ferrooxidans*), do not have higher P_U_ in HEGs than LEGs (Wilcoxon rank sum test: *P* = 0.706, two-tailed test; Table 2). These four species, being GC-rich, have few UAA-ending genes; this is consistent with our previous interpretation from Figure 2 and Figure 3, that UAA-ending genes are the main driver for increased +4U. Few UAA-ending genes implies little selection driving up +4U usage.

We investigated how stop codon and +4 nucleotide usage change with I_TE_ (a proxy of translation efficiency and gene expression) for three species (*E. coli*, *B. subtilis*, and *D. vulgaris*) that appear to represent the three different patterns: (1) +4U is overrepresented in HEGs, (2) +4U is overrepresented in only UAA-ending HEGs, and (3) +4U is not overrepresented, respectively. We binned all nonpseudo, nonhypothetical CDSs into 10 gene groups ranked by I_TE_. I_TE_ is significantly and positively correlated with P_UAA_ in all three species (*E. coli*: *R*^2^ = 0.935, *P* < 0.0001; *B. subtilis*: P_UAA_: *R*^2^ = 0.884, *P* < 0.0001; *D. vulgaris*: *R*^2^ = 0.644, *P* = 0.00518; Figure 4), even when UAA accounts for a small fraction of the stop codons. This is consistent with a previous study (Wei *et al.* 2016) showing UAA to be always preferred by HEGs. Furthermore, I_TE_ was significantly positively correlated with P_U_ in *E. coli* (*R*^2^ = 0.9149, *P* < 0.0001) and in *B. subtilis* (*R*^2^ = 0.773, *P* < 0.001), but not in *D. vulgaris* (*R*^2^ = 0.0098, *P* = 0.786). No significant relationship between other nucleotides at the +4 site and I_TE_ was observed (Figure 4). To show that the U bias exists only at the +4 site, we randomly shuffled 20 nucleotides in the (5′-UTR) for all 4140 nonpseudo, nonhypothetical *E. coli* genes, and the significant correlation between ITE and P_U_ disappeared (*R*^2^ = 0.0301, *P* = 0.632; Figure S1 in File S2). To validate that other metrics of codon usage bias return compatible results, we measured HEGs and LEGs by CAI (Table S1 in File S2); the two metrics (CAI and ITE) return similar +4U usage (Wilcoxon rank sum test with continuity correction: *P* = 0.845, two-tailed test).

The overuse of +4U in UAA-ending genes is also visible in the highly-expressed 30S and 50S ribosomal protein genes (Figure 5A, Spearman rank correlation = 0.8385, d.f. = 25, *P* < 0.0001), and the fitted nonlinear curve (Figure 5A) accounts for 82.93% of the variation in P_U.UAA_. There is no significant correlation between P_U.UAG_ and P_UAG_ (Figure 5B, *R*^2^ = 0.0032, *P* = 0.8237), and a negative linear correlation between P_U.UGA_ and P_UGA_ (Figure 5C, *R*^2^ = 0.414, *P* = 0.003968). Here, all 25 species (Figure 1) were analyzed since ribosomal protein genes were considered. To alleviate the issue of data dependence due to shared ancestry between species (Figure 1), we performed linear regression on Felsenstein’s phylogeny-based independent contrasts (Felsenstein 1985); and the correlation between P_U.UAA_ and P_UAA_ was still significant (*R*^2^ = 0.5819, *P* < 0.0001), and the result is consistent with bootstrapped trees or the tree reconstructed by using PhyPA (Xia 2016).

### Relationship between +4U usage and nc_tRNA abundance

We have hypothesized that +4U reduces misreading of stop codons, especially UAA, by nc_tRNAs (Table 1). We used tRNA gene copy numbers as a proxy of tRNA abundance. This approach has been fruitful in a number of studies (Duret and Mouchiroud 1999; Kanaya *et al.* 1999; Percudani *et al.* 1997; Chithambaram *et al.* 2014a,b; Prabhakaran *et al.* 2014). We denoted nc_tRNA1, nc_tRNA2, and nc_tRNA3 as the number of nc_tRNAs with a single mismatch at the first, second, and third stop codon site, respectively. In each species, P_nc_tRNA1_ was calculated as the number of nc_tRNA1 copies divided by the total number of tRNA copies. In the 19 bacterial species, P_U_ in UAA-ending HEGs was significantly and positively correlated with P_nc_tRNA_, the relationship being particularly strong in nc_tRNAs with a single mismatch at the first stop codon site (Figure 6). This positive correlation remains highly significant even after excluding nc_tRNA^Gln^, which is a key contributor to UAA read-through (Blanchet *et al.* 2014; Roy *et al.* 2015, 2016) (*R*^2^ = 0.517, *P* = 0.0005, Figure 6D). The correlation between P_U_ and P_nc_tRNA_ was, however, not significant in UAG and UGA-ending HEGs (Figure S2 in File S2).

To alleviate data dependence due to shared ancestry, we performed regression on independent contrasts (Felsenstein 1985) that showed significant correlation between P_U_ and P_nc_tRNA1_ (*R*^2^ = 0.349, *P* = 0.00985) and between P_U_ and P_nc_tRNA1 – Gln_ (*R*^2^ = 0.501, *P* = 0.00101), but weak linear correlation between P_U_ and P_nc_tRNA2_ (*R*^2^ = 0.150, *P* = 0.112) and P_nc_tRNA3_ (*R*^2^ = 0.233, *P* = 0.0424).

## Discussion

UAA is consistently the preferred stop codon in HEGs in a diverse array of bacterial species (Wei *et al.* 2016), presumably because: (1) UAA can be decoded by both RF1 and RF2 (Scolnick *et al.* 1968; Milman *et al.* 1969; Nakamura *et al.* 1996), and (2) UAA has the least termination read-through (Parker 1989; Jorgensen *et al.* 1993; Meng *et al.* 1995; Cesar Sanchez *et al.* 1998; Tate *et al.* 1999; Dabrowski *et al.* 2015). Our study advanced these studies by showing that: (1) +4U is strongly associated with UAA in HEGs relative to LEGs, (2) +4U usage increases with an increasing number of nc_tRNAs, and (3) both UAA and +4U usage increases with gene expressed measured by I_TE_. Taken together, these findings suggest that +4U may enhance the UAA stop signal by reducing misreading by nc_tRNAs. This interpretation is consistent with read-through studies discussed previously and with the finding that termination suppression of stop codons was least efficient in the presence of +4U in *E. coli* (Kopelowitz *et al.* 1992). Consequently, the tetranucleotide UAAU is expected to represent the strongest termination signal a variety of bacterial lineages.

The interpretation above also explains why +4U is not overused in GC-rich species (Figure 3 and Table 2), because these species have few genes ending with UAA. If +4U mainly enhances the termination signal of UAA against misreading by nc_tRNAs, the rarity of UAA-ending genes is naturally expected not to associate with overuse of +4U.

The importance of considering gene expression (or translation efficiency) in studying codon adaptation is highlighted by the fact that little +4U bias would be observed in the 19 species when all CDSs were considered (Figure S3 in File S2) without contrasting between HEGs and LEGs. It is also important to study +4U bias separately for different stop codons because nucleotide distribution at the +4 site is heterogeneous among genes ending with different stop codons (Figure 2). Previous studies on termination read-through in yeast (Roy *et al.* 2015, 2016) and bacteria (Kramer and Farabaugh 2007) often did not take into consideration all of the possible combinations of stop codons and the +4U nucleotide.

Our study also suggests phylogenetic inertia in the evolution of the stop codon decoding mechanism. For example, all five species in Betaproteobacteria exhibit very similar differences between HEGs and LEGs in +4U usage, so do the seven species belonging to the supercluster including Cyanobacteria and Bacilli (Figure 1). For this reason, phylogeny-based comparative methods are crucial for the proper assessment of statistical significance among variables.

It is interesting to note that UGA- and UAG-ending genes do not show the same strong preference for +4U observed in UAA-ending genes. Given that RF1 decodes UAA and UAG, and RF2 decodes UAA and UGA, it seems that RF1 and RF2 must have different binding dynamics between UAA- and UAG-ending genes. Structural (Matheisl *et al.* 2015; Svidritskiy *et al.* 2016; Tang *et al.* 2016) or cross-linking studies (Brown and Tate 1994; Tate *et al.* 1996; Poole *et al.* 1997, 1998) may shed light on the effect of +4U on the UAA termination signal.

## 

## Supplementary Material

Supplemental material is available online at www.genetics.org/lookup/suppl/doi:10.1534/genetics.116.193961/-/DC1.
