# Supplementary material for: The Role of +4U as an Extended Translation Termination Signal in Bacteria
Source: Genetics. 2016 Nov 29;205(2):539–49. doi: 10.1534/genetics.116.193961 (PMC5289835; doi:10.1534/genetics.116.193961)
Supplement: Supplementary file 1 [file 539FileS1.pdf]

Description of supplemental files S2, S3, S4 and S5

S2. Supplementary Table and figures discussed in the manuscript, with figure legends.

S3. RSCU data of 25 bacterial species were obtained in DAMBE: "Seq. Analysis| Codon Usage | Relative Synonymous Codon Usage" for 40 ribosomal protein genes with highest protein abundances and 40 genes with lowest non-zero protein abundances as reference HEGs and LEGs, respectively. All genes selected are non-pseudo, non-hypothetical. AA denotes amino acid coded, CF_LEG denotes the total codon counts. H_RSCU denotes the RSCU values in HEGs computed in DAMBE. The codons are separated as R-ending, Y-ending and others. The RSCU values are plotted for R-ending codons and others, not Y-ending codons (never show a slop of RSCU_HEG> RSCU_LEG due to the synonymous pair decoded by the same tRNA).

S4. ITE values for all non-pseudo, non-hypothetical coding DNA sequences in 19 bacterial species. ITE was computed for each species using their respective .ITE reference files compiled using CF_HEG and CF_LEG in supplemental file S3, in DAMBE: "Seq. Analysis| Codon Usage| Index of translation elongation (I_TE)". The method is "Break 8-fold and 6-fold families into 2". Stop codons were obtained in DAMBE: "Sequence| Sequence Manipulation| Keep rightmost sites: 3". The +4 base was obtained in DAMBE: "File| Open standard sequence file| Downstream of CDS: 1".

S5. Data for figures 2 to 6 in the manuscript.
