# Supplementary material for: The Role of +4U as an Extended Translation Termination Signal in Bacteria
Source: Genetics. 2016 Nov 29;205(2):539–49. doi: 10.1534/genetics.116.193961 (PMC5289835; doi:10.1534/genetics.116.193961)
Supplement: Supplementary file 2 [file 539FileS2.pdf]

Table S1. The usage of +4U in 100 non-pseudo, non-hypothetical HEGs and LEGs for each stop codon, ranked by CAI, in 25 bacterial species. Together with the number of 30S and 50S ribosomal protein genes in each species. The reference HEGs to compute CAI are the same 40 ribosomal protein genes used for .ITE reference files.

			UAA[b]		UAG		UGA	
SPECIES NAME	ACCESSION	N.R[a]	$P_{U.HEG}$	$P_{U.LEG}$	$P_{U.HEG}$	$P_{U.LEG}$	$P_{U.HEG}$	$P_{U.LEG}$
Bacillus anthracis	NC_005945	56	0.77	0.23	0.37	0.29	0.32	0.24
Bacillus subtilis	NC_000964	56	0.65	0.24	0.19	0.10	0.44	0.34
Bartonella henselae	NC_005956	55	0.25	0.35	0.21	0.19	0.39	0.35
Staphylococcus aureus	NC_002758	53	0.78	0.35	0.34	0.32	0.49	0.343
Listeria monocytogenes	NC_003210	57	0.52	0.29	0.17	0.23	0.36	0.36
Streptococcus pyrogenes	NC_002737	51	0.61	0.23	0.34	0.24	0.51	0.49
Lactococcus lactis	NC_002662	57	0.64	0.17	0.36	0.34	0.32	0.32
Leptospira interrogans	NC_005823	54	0.37	0.26	0.30	0.34	0.41	0.33
Deinococcus deserti	NC_002937	51	0.17	0.14	0.12	0.11	0.12	0.17
Bacteriodes thetaiotaomicron	NC_004663	55	0.62	0.29	0.34	0.21	0.46	0.28
Escherichia coli	NC_000913	57	0.62	0.3	0.36	0.24	0.72	0.30
Salmonella enterica	NC_003197	59	0.60	0.35	0.36	0.25	0.69	0.30
Yesinia pestis	NC_003143	56	0.63	0.39	0.33	0.31	0.65	0.45
Shewanella oneidensis	NC_004347	62	0.71	0.27	0.31	0.27	0.49	0.328

Neisseria meningitidis	NC_003112	57	0.52	0.21	0.33	0.32	0.59	0.27
Legionella pneumophila	NC_002942	54	0.37	0.39	0.29	0.29	0.42	0.26
Acidithiobacillus ferrooxidans	NC_011761	56	0.15	0.30	0.15	0.20	0.24	0.31
Campylobacter jejuni	NC_002163	54	0.45	0.30	0.29	0.28	0.34	0.43
Desulfovibrio vulgaris	NC_002937	56	0.19	0.18	0.14	0.11	0.37	0.21
Mycobacterium tuberculosis	NC_000962	58	0.17	0.14	0.16	0.17	0.20	0.24
Microcystis aeruginosa	NC_010296.1	53	0.39	0.19	0.34	0.37	0.35	0.31
Helicobacter pylori	NC_000915	54	0.21	0.20	0.22	0.34	0.41	0.45
Pseudomonas aeruginosa	NC_002516	58	0.21	0.22	0.11	0.13	0.37	0.23
Shigella flexneri	NC_004337	53	0.62	0.17	0.47	0.20	0.63	0.37
Synechocystis sp.	NC_017277	52	0.32	0.26	0.35	0.18	0.38	0.33

a Number of 30S and 50S ribosomal protein genes.
b The +4U usage in 100 UAA-ending HEGs and LEGs (highest and lowest CAI scores, respectively).

FIGURE LEGENDS

Fig. S1. Relationship between I_{TE} and usage of termination signals (stop codons and +4 bases), in *E. coli*. All non-pseudo, non-hypothetical CDSs were ranked by I_{TE} and binned into 10 sets. Twenty sequences in the 5' UTR were randomly shuffled using Sequence Manipulation Suite: Shuffle DNA (Stothard 2000). The stop codon usage and +4 base usage was obtained in each set. Stop codon usage (P_{UAA}, P_{UAG}, P_{UGA}) is represented by solid lines; +4 base usage (P_A, P_C, P_G, P_U) is represented by dotted lines.

Fig. S2. Relationship between nc_tRNA abundance and +4U usage, represented by linear regression between 100 UAA-ending HEGs (highest I_{TE} scores) and abundance of UAG nc_tRNAs with a single mismatch at A) the first stop codon site, B) the second stop codon site, C) the third stop codon site, and abundance of UGA nc_tRNAs with a single mismatch at D) the first stop codon site, E) the second stop codon site, F) the third stop codon site.

Fig. S3. The +4 base usage in all non-pseudo, non-hypothetical CDSs in 19 bacterial species (Table 1).

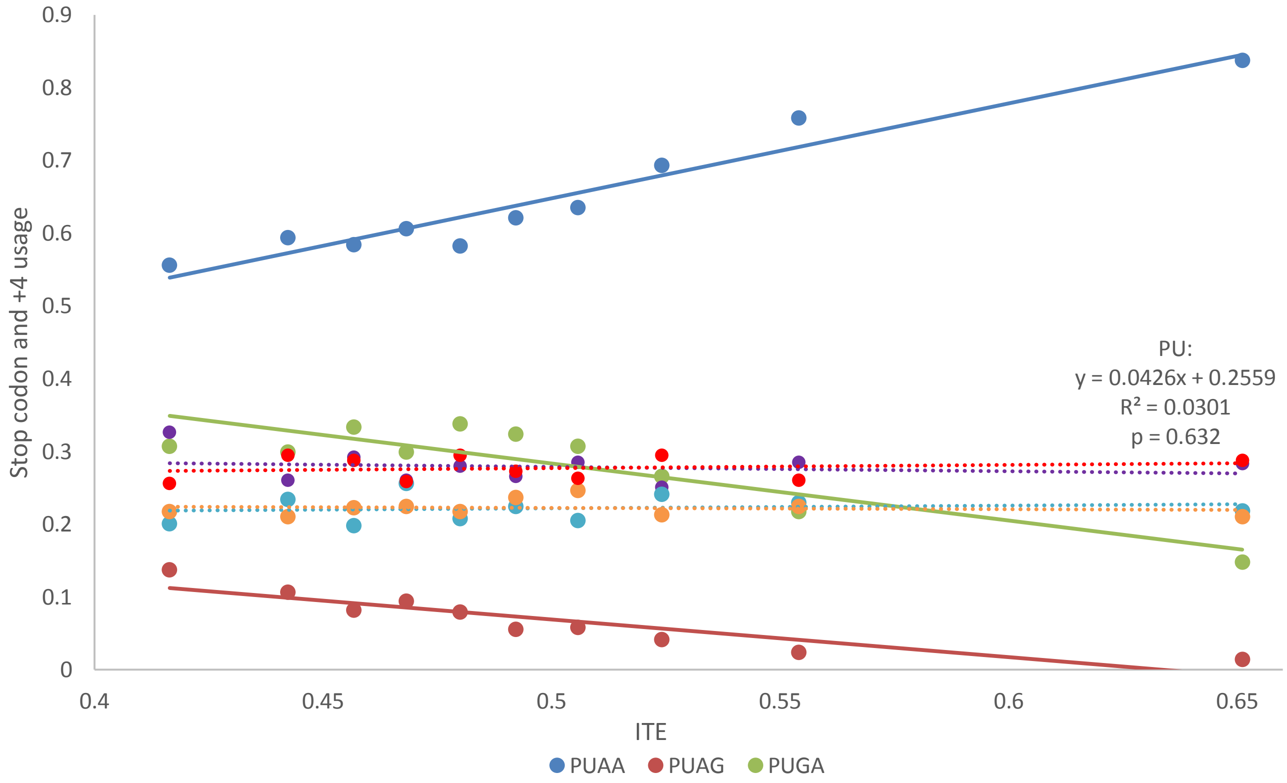
0.9
0.8
0.7
0.6
0.5
0.4
0.3
0.2
0.1
0
Stop codon and +4 usage
PU:
y = 0.0426x + 0.2559
R² = 0.0301
p = 0.632
0.4
0.45
0.5
0.55
0.6
0.65
ITE
PUAA
PUAG
PUGA

Fig. S1

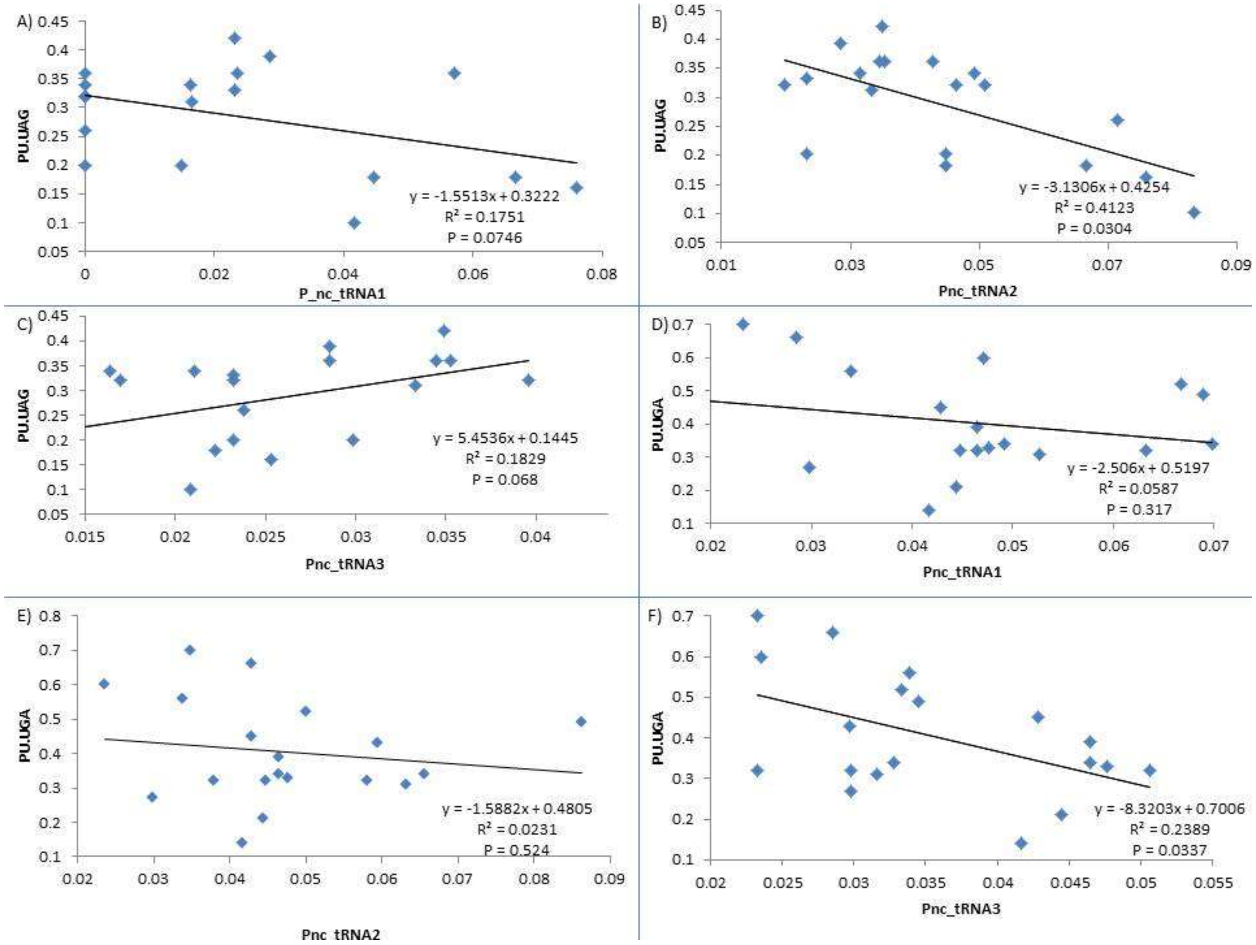
A)
PU.UAG
P_nc_tRNA1
y = -1.5513x + 0.3222
R² = 0.1751
P = 0.0746
B)
PU.UAG
Pnc_tRNA2
y = -3.1306x + 0.4254
R² = 0.4123
P = 0.0304
C)
PU.UAG
Pnc_tRNA3
y = 5.4536x + 0.1445
R² = 0.1829
P = 0.068
D)
PU.UGA
Pnc_tRNA1
y = -2.506x + 0.5197
R² = 0.0587
P = 0.317
E)
PU.UGA
Pnc tRNA2
y = -1.5882x + 0.4805
R² = 0.0231
P = 0.524
F)
PU.UGA
Pnc_tRNA3
y = -8.3203x + 0.7006
R² = 0.2389
P = 0.0337

Fig. S2

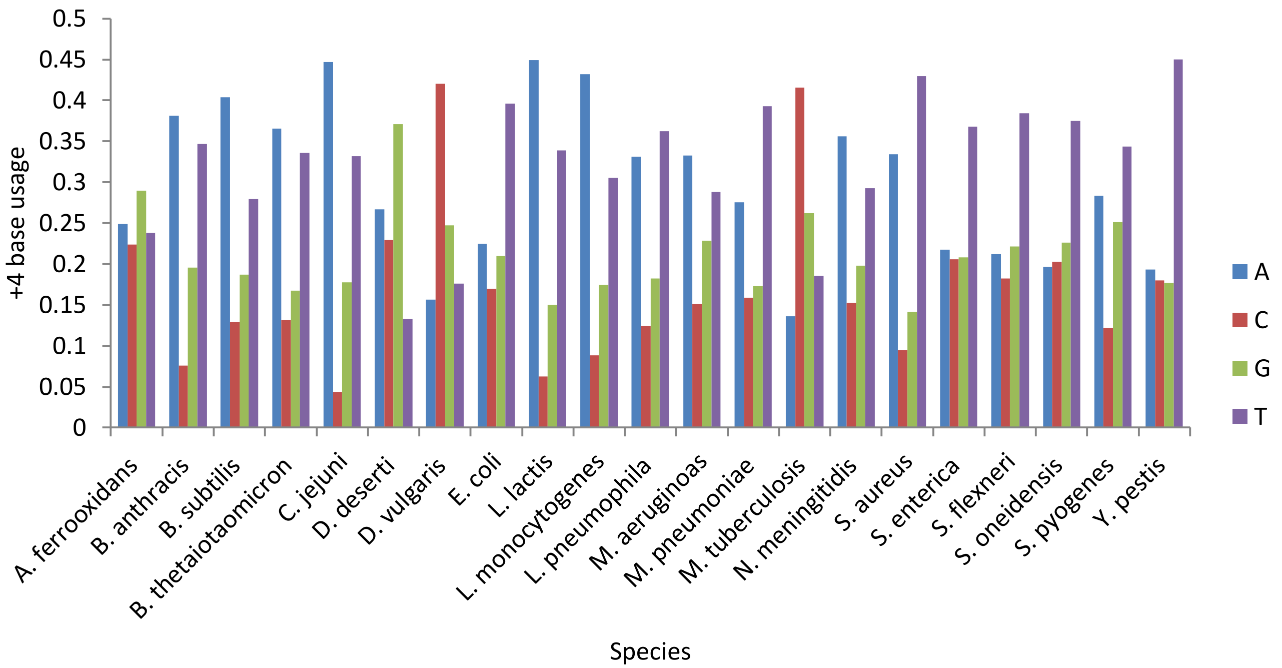

0.5
0.45
0.4
0.35
0.3
0.25
0.2
0.15
0.1
0.05
0
+4 base usage
A
C
G
T
A. ferrooxidans
B. anthracis
B. subtilis
B. thetaiotaomicron
C. jejuni
D. deserti
D. vulgaris
E. coli
L. lactis
L. monocytogenes
L. pneumophila
M. aeruginoas
M. pneumoniae
M. tuberculosis
N. meningitidis
S. aureus
S. enterica
S. flexneri
S. oneidensis
S. pyogenes
Y. pestis
Species

Fig. S3
