# Supplementary material for: The Role of +4U as an Extended Translation Termination Signal in Bacteria
Source: Genetics. 2016 Nov 29;205(2):539–49. doi: 10.1534/genetics.116.193961 (PMC5289835; doi:10.1534/genetics.116.193961)
Supplement: Supplementary file 3 [file 539FileS3.docx]

File S3: RSCU data of 25 bacterial species were obtained in DAMBE: “Seq. Analysis| Codon Usage | Relative Synonymous Codon Usage” for 40 ribosomal protein genes with highest protein abundances and 40 genes with lowest non-zero protein abundances as reference HEGs and LEGs, respectively. (.xlsx, 307 KB)

Available for download as a .xlsx file at:

http://www.genetics.org/lookup/suppl/doi:10.1534/genetics.116.193961/-/DC1/FileS3.xlsx
