# Supplementary material for: The Role of +4U as an Extended Translation Termination Signal in Bacteria
Source: Genetics. 2016 Nov 29;205(2):539–49. doi: 10.1534/genetics.116.193961 (PMC5289835; doi:10.1534/genetics.116.193961)
Supplement: Supplementary file 5 [file 539FileS4.docx]

File S4: ITE values for all non-pseudo, non-hypothetical coding DNA sequences in 19 bacterial species. (.xlsx, 1 MB)

Available for download as a .xlsx file at:

http://www.genetics.org/lookup/suppl/doi:10.1534/genetics.116.193961/-/DC1/FileS4.xlsx
