# Supplementary material for: The Role of +4U as an Extended Translation Termination Signal in Bacteria
Source: Genetics. 2016 Nov 29;205(2):539–49. doi: 10.1534/genetics.116.193961 (PMC5289835; doi:10.1534/genetics.116.193961)
Supplement: Supplementary file 7 [file 539FileS5.docx]

File S5: Data for figures 2 to 6 in the manuscript. (.xlsx, 310 KB)

Available for download as a .xlsx file at:

http://www.genetics.org/lookup/suppl/doi:10.1534/genetics.116.193961/-/DC1/FileS5.xlsx
